# Efficacy and Safety of Sofosbuvir-Based Direct-Acting Antiviral Agents Treatment for Patients with Genotype 3/6 Hepatitis C Virus Infection

**DOI:** 10.1155/2020/8872120

**Published:** 2020-10-31

**Authors:** Yong-yu Mei, You-ming Chen, Yuan-kai Wu, Xiao-hong Zhang, Wen-xiong Xu

**Affiliations:** ^1^Department of Infectious Diseases, Third Affiliated Hospital of Sun Yat-Sen University, Guangzhou 510630, Guangdong, China; ^2^Guangdong Key Laboratory of Liver Disease Research, Third Affiliated Hospital of Sun Yat-Sen University, Guangzhou 510630, Guangdong, China

## Abstract

**Aims:**

The aim is to evaluate the efficacy and safety of Sofosbuvir- (SOF-) based direct-acting antiviral agents (DAAs) treatment for patients with genotype (GT) 3/6 hepatitis C virus (HCV) infection.

**Methods:**

Patients infected with GT 3/6 HCV and treated with SOF-based DAAs were enrolled in this prospective, open, single-center, and real-world study. Drugs included Sofosbuvir (SOF), Velpatasvir (VEL), Daclatasvir (DCV), and Ribavirin (RBV). The treatment regimens included SOF + RBV for 24 weeks, SOF + DCV ± RBV for 12/24 weeks, and SOF/VEL ± RBV for 12 weeks.

**Results:**

A total of 54 patients were included. Age was 42.5 ± 10.4 years. Baseline HCV RNA was 6.29 ± 0.89log10 IU/mL. The numbers of GT 3a, 3b, and 6a patients were 10, 12, and 32, respectively. The numbers of chronic hepatitis, compensated cirrhosis, and decompensated cirrhosis patients were 39, 9, and 6, respectively. In patients with chronic hepatitis C and liver cirrhosis, sustained virological response 12 weeks after the end of treatment (SVR12) was 97.4% and 96.7%, respectively, and rapid virological response (RVR) was 75.0% and 57.1%, respectively. SVR12 of GT3a, GT3b, and GT6a was 100%, 83.3%, and 97%, respectively. ALT normality rate in chronic hepatitis group is higher than that in cirrhosis group at 4 weeks of treatment (89.7% versus 60.0%, *p* = 0.033) and at 12 weeks after EOT (94.9% versus 66.7%, *p* = 0.021). The overall incidence rate of adverse events was 44.4%, with fatigue being the most common (13.0%).

**Conclusion:**

SOF-based DAAs regimen can achieve ideal SVR12 for Chinese patients with both GT3a and GT6a HCV infection. The tolerance and safety of SOF-based DAAs regimen are good.

## 1. Introduction

There are 71 million chronic hepatitis C (CHC) patients worldwide [1–3], and 350,000 people die of hepatitis C-related diseases every year. In China, 10 million people are infected with hepatitis C virus (HCV) [4]. HCV infection has become a serious public health problem.

Genotype 3 (GT3) and GT6 HCV infections are more common in southeast Asia and south China [5, 6]. People with these two genotypes are at higher risk of developing liver cancer [4, 7–9]. Compared with other genotypes of chronic HCV infection, patients with GT3-CHC have a faster progression in liver disease [7]. Direct-acting antiviral agents (DAAs) are the first choice for HCV by Chinese Medical Association [4], American Association for the Study of Liver Diseases [10], and European Association for the Study of the Liver [11]. The treatment regimens include multiple solutions based on Sofosbuvir (SOF). Compared with GT1-CHC, GT3-CHC patients have a relatively low sustained virological response 12 weeks after the end of treatment (SVR12) by DAAs [12]. Satisfactory SVR12 has been achieved for DAAs treatment in GT6-CHC patients, but the number of patients in clinical trials is limited. Otherwise, there is still lack of data on the efficacy and safety of DAAs treatment in Chinese population in real-world study.

Therefore, we design this prospective and real-world study and aim to compare the efficacy and safety of different DAAs treatments based on SOF for CHC patients with GT 3/6 in China. We hope the results can provide supplementary data for clinical medication.

## 2. Materials and Methods

### 2.1. Study Design and Subjects

This was a prospective, open, single-center, and real-world study. Patients, who were infected with GT 3/6 HCV and treated with DAAs based on SOF in Third Affiliated Hospital of Sun Yat-sen University from December 1, 2017, to September 30, 2019, were enrolled in the study. This study complied with the Declaration of Helsinki and was approved by the ethics committee of the Third Affiliated Hospital of Sun Yat-sen University. The ethical approval number was [2018] 02-305-02. All participants provided written informed consent prior to enrollment in the study.

Enrollment criteria were the following: (1) CHC patients [4], (2) age of 18–65 years, (3) GT3 or GT6 HCV infection, (4) treatment with DAAs based on SOF, and (5) voluntarily signing informed consent form and participating in the follow-up cohort of hepatitis C in Third Affiliated Hospital of Sun Yat-sen University.

Exclusive criteria were the following: (1) patients with renal disfunction (eGFR < 30 mL/min/1.73 m^2^), (2) patients taking drugs that had interactions with DAAs, and (3) patients with autoimmune diseases or those who acquired immune deficiency syndrome.

### 2.2. Treatment Regimens

Drugs included Sofosbuvir (SOF, oral 400 mg per day), Velpatasvir (VEL, oral 100 mg per day), Daclatasvir (DCV, oral 60 mg per day), and Ribavirin (RBV, oral 10∼15 mg/kg per day). SOF (Sovaldi, GILEAD) was officially launched in China on October 21, 2017. SOF/VEL (Epclusa, GILEAD) was officially launched in China on May 30, 2018. DCV (Daklinza, Bristol-Myers Squibb) was officially launched in China on August 24, 2017. Patients can obtain these drugs about 2 months thereafter.

According to guideline [4, 10, 11] of HCV treatment, drug instructions, and drug accessibility in China, the treatment regimens included SOF + RBV for 24 weeks, SOF + DCV ± RBV for 12/24 weeks, and SOF/VEL ± RBV for 12 weeks. RBV was used in singular DAA regimen of SOF for chronic hepatitis and cirrhosis patients or in SOF-based DAAs regimens for cirrhosis patients.

### 2.3. Follow-Up

The patients were followed up at baseline, 4 weeks of treatment, 8 weeks of treatment, end of treatment (EOT), 12 weeks after EOT, and 24 weeks after EOT. At each follow-up, patient's data, including symptoms, signs, and laboratory tests, were recorded. Laboratory tests included blood cells test (white blood cells, red blood cells, hemoglobin, and platelets), blood biochemical test (aspartate aminotransferase [AST], alanine aminotransferase [ALT], total bilirubin [TBil], blood urea nitrogen [BUN], and creatinine), virological test (anti-hepatitis A virus immunoglobulin M [HAV-IgM], hepatitis B virus surface antigen [HBsAg], HBV DNA, anti-HCV immunoglobulin G [HCV-IgG], HCV RNA, and anti-hepatitis E virus immunoglobulin M and G [HEV-IgM and HEV-IgG]), liver ultrasound, and Fibroscan.

Routine automated techniques were used for all biochemical tests at our clinical laboratories. Serum HBV DNA levels were measured with real-time PCR using the COBAS AmpliPrep/COBAS TaqMan HBV Test, version 2.0 (detection limit: 20 IU/mL, Roche Molecular Systems, Inc.). Serum HCV RNA levels were measured with real-time PCR using the COBAS AmpliPrep/COBAS TaqMan HCV Test, version 2.0 (detection limit: 15 IU/mL, Roche Molecular Systems, Inc.).

### 2.4. Definition of Virological Response

Rapid viral response (RVR) is defined as HCV RNA being undetectable after 4 weeks of DAAs treatment. Early viral response (EVR) is defined as HCV RNA being undetectable after 8 weeks of DAAs treatment. End of treatment response (EOTR) is defined as HCV RNA being undetectable at the end of DAAs treatment. Sustained virological response 12 weeks after EOT (SVR12) is defined as HCV RNA being undetectable 12 weeks after the end of DAAs treatment.

### 2.5. Statistical Analysis

Continuous data were indicated with mean ± standard deviation (SD), while categorical data were reported as count and percentage (%). Student's independent *t*-test and one-way ANOVA were used to test the difference of means between 2 groups or among multiple groups (>2). Nonparametric tests including the Mann-Whitney *U* test and Kruskal-Wallis test were used to compare means between groups for data normality was not assumed. Categorical data were tested with Chi-square test or Fisher's exact test (if any expected value ≤ 5 was found). The statistical significance level for all the tests was set at a *p* value < 0.05. Statistical analyses were performed using IBM SPSS Version 20 (SPSS Statistics V20, IBM Corporation, Somers, New York).

## 3. Results

### 3.1. Patients Baseline Characteristics

**Figure 1:**
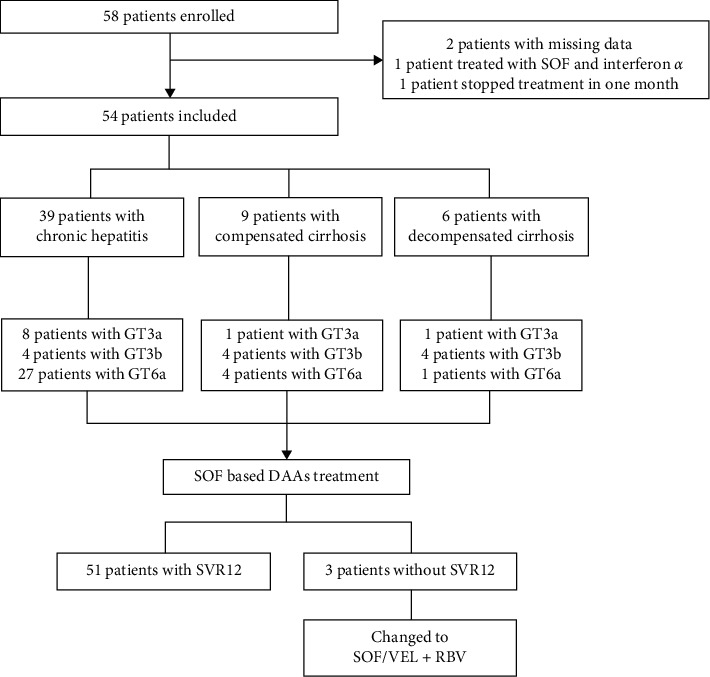
The CONSORT diagram of patient enrollment.

A total of 58 CHC patients with GT3/6 were enrolled in this prospective study. Two cases had missing data: 1case was treated with SOF and interferon *α*, and 1 case stopped treatment in one month. Therefore, a total of 54 patients were finally included in this study. The CONSORT diagram of patient enrollment is shown in Figure 1.

The average age of the 54 patients was 42.5 ± 10.4 years. Forty-two patients were males, while 12 were females. There were 39, 9, and 6 patients with chronic hepatitis, compensated cirrhosis, and decompensated cirrhosis, respectively. In patients with chronic hepatitis, there were 8, 4, and 27 patients with GT3a, GT3b, and GT6a, respectively. In patients with compensated cirrhosis, there were 1, 4, and 4 patients with GT3a, GT3b, and GT6a, respectively. In patients with decompensated cirrhosis, there were 1, 4, and 1 patient with GT3a, GT3b, and GT6a, respectively. In total, there were 10, 12, and 32 patients with GT3a, GT3b, and GT6a, respectively. Baseline level of HCV RNA was 6.29 ± 0.89 log10 IU/mL. The duration from diagnosis to treatment was 40.2 ± 64.4 months. The duration of follow-up was 46.1 ± 23.5 weeks. The demographic information and baseline characteristics of the patients are shown in Table 1.

### 3.2. Safety of SOF-Based DAAs Treatment

The overall incidence of adverse events (AEs) is 44.4% (24/54), including 7 (12.9%) patients with fatigue, 3 (5.6%) patients with rash, 2 (3.7%) patients with itchy skin, 2 (3.7%) patients with headache and dizziness, 1 (1.9%) patient with gastric ulcer, 1 (1.9%) patient with myalgia, 1 (1.9%) patient with prolonged menstruation, and 1 (1.9%) patient with abdominal pain and diarrhea. The laboratory tests abnormalities mainly included 2 (3.7%) patients with elevated TBil, 2 (3.7%) patients with decreased hemoglobin, and 1 (1.9%) patient with decreased platelets. Interestingly, 5 out of 6 (83.3%) patients in decompensated cirrhosis group had AEs. Most of AEs disappeared after proper treatment, unless serious adverse events (SAEs) occurred in 1 patient, who was diagnosed with hepatocellular carcinoma (HCC) at EOT with pathological evidence and received liver cancer resection. Seven patients were coinfected with hepatitis B virus (HBV), and none of them had HBV reactivation in DAAs treatment. One patient had a history of liver transplantation for 5 years, and no AEs occurred in DAAs treatment. None of the 54 patients stopped DAAs treatment due to AEs, and none of them died from AEs in DAAs treatment course.

### 3.3. Efficacy of SOF-Based DAAs Treatment

**Figure 2:**
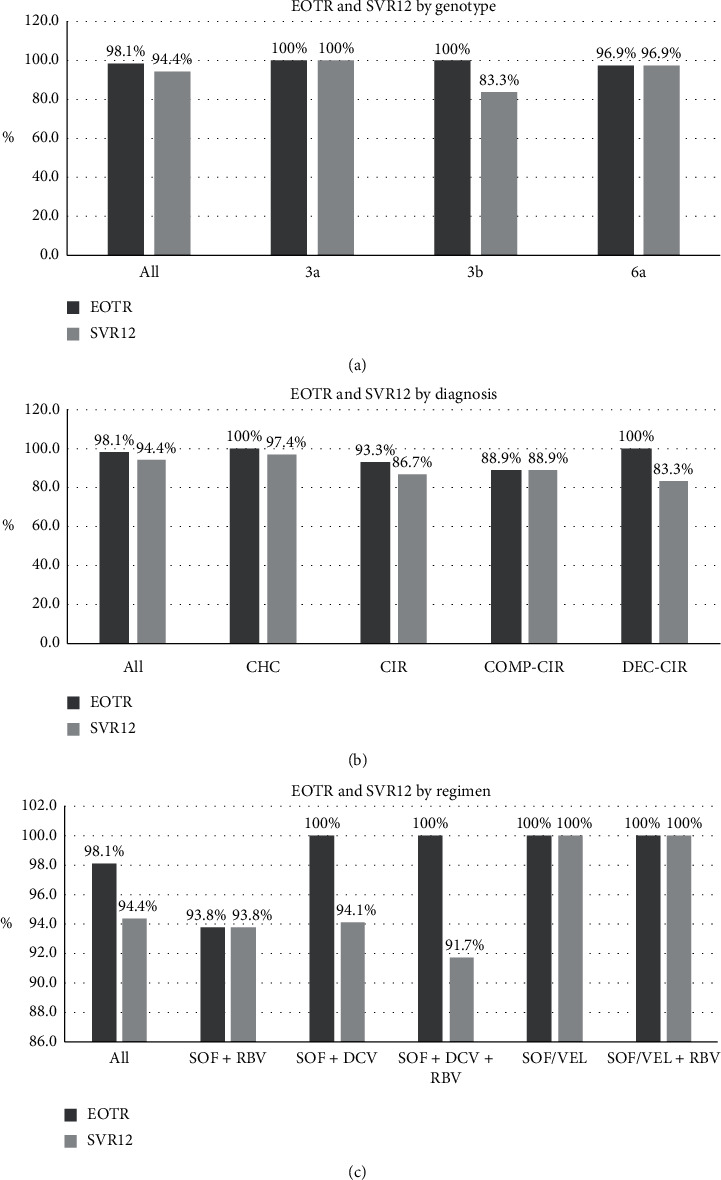
EOTR and SVR12. (a) EOTR and SVR12 in patients with different genotype. (b) EOTR and SVR12 in patients with different diagnosis. (c) EOTR and SVR12 in patients with different treatment plan.

Baseline level of HCV RNA was 6.33 ± 0.81 and 6.17 ± 1.11 log10 IU/mL in patients of chronic hepatitis and cirrhosis, respectively (*p* > 0.05). After SOF-based DAAs treatment, RVR was 75.0% and 57.1% in patients of chronic hepatitis and cirrhosis, respectively. EVR was 100% and 93.3% in patients of chronic hepatitis and cirrhosis, respectively. EOTR was 100% and 93.3% in patients of chronic hepatitis and cirrhosis, respectively. SVR12 was 97.4% and 96.7% in patients of chronic hepatitis and cirrhosis, respectively. The results are shown in Table 2. For patients with different genotype, EOTR and SVR12 are shown in Figure 2(a). For patients with different diagnosis (chronic hepatitis, compensated cirrhosis, and decompensated cirrhosis), EOTR and SVR12 were shown in Figure 2(b). For patients with different treatment plan, EOTR and SVR12 were shown in Figure 2(c).

There were 3 patients without SVR12. Among them, Patient A had CHC with GT3b and was treated with SOF and DCV for 12 weeks; Patient B had compensated cirrhosis with GT6a and was treated with SOF and RBV for 24 weeks; Patient C had decompensated cirrhosis with GT3b and was treated with SOF, DCV, and RBV for 24 weeks. Then, as they did not achieve SVR12, Patients A and C changed to be treated with SOF/VEL + RBV for 24 weeks, and Patient B changed to be treated with SOF/VEL + RBV for 12 weeks. All of them achieved SVR12 after that.

For all the 54 patients, baseline ALT abnormality rate was 66.7%. ALT normality rate in chronic hepatitis group is higher than that in cirrhosis group at 4 weeks of treatment (89.7% versus 60.0%, *p* < 0.05) and at 12 weeks after EOT (94.9% versus 66.7%, *p* < 0.05). Baseline level of TBil in cirrhosis group is higher than that in chronic hepatitis group. But there were not statistical differences of the two groups in TBil changes in the treatment course or the follow-up thereafter (all *p* > 0.05). Meanwhile, there were no statistical differences of the two groups in estimated glomerular filtration rate (eGFR) changes in the treatment course or the follow-up thereafter (all *p* > 0.05). The biochemical response of DAAs treatment is shown in Table 3.

## 4. Discussion

DAAs were officially launched in China in 2017, but there have not been many real-world studies of DAAs treatment in GT3 and GT6 HCV infection in China so far. In our study, the SOF-based DAAs regimen could achieve SVR12 in 94.4% of all the patients included. SVR12 was 100% in patients with GT3a, 97% in patients with GT6a, and 83.3% in patients with GT3b. Otherwise, the overall safety of the SOF-based DAAs regimen was good.

Our study showed that AEs of the SOF-based DAAs regimen were mild. AEs were mainly fatigue and rash. Symptoms relieved after proper treatment, and drug withdrawal was not needed. Abnormal laboratory data, mainly including increased TBil and decreased HGB and PLT, occurred in cirrhosis patients, especially in decompensated ones. The decreased HGB and PLT could recover to normal level after adjusting the dosage of ribavirin. However, the mechanism of PLT decline was unclear. Although patients with HBV coinfection in this study did not have elevated level of HBV DNA, we found cases of HBV activation after SVR in our previous study [13]. The progression of liver disease to cirrhosis and hepatocellular carcinoma is generally faster in CHC patients who are coinfected with HBV, and HCV is usually more predominant. Immunosuppression of the host or eradication of hepatitis C can change this paradigm, causing hepatitis B reactivation [14]. Therefore, CHC patients with HBV coinfection still needed to monitor level of HBV DNA after DAAs treatment. Intrahepatic occupancy was not found at baseline, but hepatocellular carcinoma was found at the end of DAAs treatment in one patient in our study. DAAs treatment can inhibit replication of HCV and achieve SVR. After that, the progression of liver disease may slow down [15]. DAAs treatment cannot directly prevent occurrence of liver cancer. However, underlying liver cirrhosis is present in most patients with HCC, the impact of liver function is relevant to establish treatment approach, and antiviral treatment could prevent worsening of liver function, allowing anti-HCC treatment [16]. Whether DAAs can predispose to HCC or not is still conflicting so far [17]. A study from Taiwan indicated that the risk of HCC recurrence and progression is not increased by DAAs [18]. In patients with HCV-related cirrhosis who had been successfully treated for early HCC, DAAs significantly improved OS compared with no DAA treatment [19]. A meta-analysis [20] showed that lower serum albumin, randomized controlled trial study design, and follow-up were independently associated with higher recurrence risk, whereas tumour size and alpha-fetoprotein levels were associated with higher mortality in patients with successfully treated HCV-related HCC.

ALT normality rate in chronic hepatitis group is higher than that in cirrhosis group at 4 weeks of treatment (89.7% versus 60.0%, *p* < 0.05) and at 12 weeks after EOT (94.9% versus 66.7%, *p* < 0.05) in our study. It may be due to the change of liver structure in cirrhosis patients. Hepatocellular inflammation may be caused by not only HCV replication but also the immune response to liver cirrhosis. eGFR did not decrease neither in the treatment course nor in the follow-up thereafter, showing good renal tolerance of SOF-based DAAs regimen. It is inconsistent with the research by Liu et al., which found that patients receiving SOF-based DAAs exhibited a quadratic trend, with eGFR worsening on treatment and improving off treatment [21].

In a large multinational CHC cohort from East Asia, oral DAAs were highly effective (the overall SVR12 was 96%) and well tolerated across the region [22]. Of the all-oral regimens, SVR12 in GT3 CHC patients was 90–95% [12]. SVR12 could achieve 100% in GT6 CHC patients with DAAs regimen in southwest China in a real-world study [23]. In our study, SOF-based DAAs regimen could achieve fast elimination of HCV in most of the CHC patients with GT3a/3b/6a. The overall RVR, EVR, EOTR, and SVR12 were 69.6%, 97.8%, 98.1%, and 94.4%, respectively. SVR of GT3 CHC patients was ideal in the era of combination treatment of peginterferon and Ribavirin before DAAs, as it reached about 70% (68.2–71.5%) and was much higher than that of GT1. However, SVR of GT3 CHC patients was not ideal in the era of SOF-based DAAs treatment, as it reached about 90% and was lower than that of other genotypes in several studies [12, 24–27]. GT3 is regarded as being more difficult to treat as it is a relatively aggressive genotype, associated with greater liver damage and cancer risk; some subgroups of patients with GT3 infection are less responsive to current licensed DAA treatments [12]. In our study, SVR12 was 83.3% with GT3b, lower than that of GT3a (100%) and GT6a (97%). SVR12 was similar in both chronic hepatitis and cirrhosis patients with GT3b (*p* > 0.05) or in both compensated and decompensated cirrhosis patients with GT3b (*p* > 0.05). Otherwise, there was a relative high incidence of liver cancer in GT3 CHC patients [8, 9]. So, it was important to choose an adequate DAAs regimen, such as SOF-based DAAs regimens combined with RBV for prolonged duration, or Pibrentasvir + Glecaprevir [28], for patients with GT3b HCV infection.

In our study, EOTR and SVR12 were 100% in CHC patients with DAAs regimen of SOF/VEL ± RBV. But SVR12 was relatively low in CHC patients with DAAs regimen of SOF + RBV and SOF + DCV ± RBV. SOF/VEL ± RBV seemed to be a prior regimen of initial treatment for GT3/6 CHC patients. Studies have shown that DAAs regimen of SOF/VEL ± RBV can achieve ideal SVR12 [29, 30]. The three patients with initial DAAs treatment failure in our study received sequential DAAs regimen of SOF/VEL + RBV and gained SVR12 after that. DAAs regimen of SOF/VEL + RBV can also be a solution for previous DAAs treatment failure [31].

There are several limitations in our study. First, the sample size is small, which may lead to statistical bias and influence the result and clinical decision. Clinical studies with large population are needed to confirm our results. Second, there are three different treatment regimens in our study. As a real-world study, treatment regimen decision is based on treatment guidelines, drug accessibility, drug price, patient compliance, and so on. The heterogeneity of patients at baseline makes it difficult to compare the advantages and disadvantages of different treatment regimens.

## 5. Conclusions

SOF-based DAAs regimen can achieve ideal SVR12 for Chinese patients with both GT3a and GT6a HCV infection. The tolerance and safety of SOF-based DAAs regimen are good. The results need confirmation on larger populations.

## Figures and Tables

**Table 1:** Demographic information and baseline characteristics.

	All (*n* = 54)	CHC (*n* = 39)	CIR (*n* = 15)	Statistics	*p*
Age (years)	42.5 ± 10.4	40.1 ± 10.0	49.3 ± 8.4	3.066	0.003
Sex (male/female)	40/14	32/7	10/5	0.727	0.394
BMI (kg/m^2^)	23.0 ± 4.0	22.6 ± 3.6	24.2 ± 4.7	1.286	0.205
Decompensated cirrhosis	6 (11.1%)	0 (0%)	6 (40.0%)	—	—
Genotype (3a/3b/6a)	10/12/32	8/4/27	2/8/5	10.789	0.005
HCV RNA (log_10_ IU/mL)	6.29 ± 0.89	6.33 ± 0.81	6.17 ± 1.11	0.988	0.328
ALT (IU/L)	91.6 ± 68.0	99.6 ± 75.6	70.0 ± 35.1	1.841	0.072
TBil (*μ*mol/L)	13.8 ± 7.4	11.2 ± 4.6	20.3 ± 9.4	3.306	0.005
Alcohol addiction	16 (29.6%)	9 (23.1%)	7 (46.7%)	1.871	0.171
Route of infection				1.132	0.568
Transfusion	8 (14.8%)	5 (12.8%)	3 (20.0%)		
IDUs	16 (29.6%)	13 (33.3%)	3 (20.0%)		
Unknown	30 (55.6%)	21 (53.8%)	9 (60.0%)		
HBV coinfection	7 (13.0%)	4 (10.3%)	3 (20.0%)	0.253	0.615
Duration from diagnosis to treatment (months)	40.2 ± 64.4	35.0 ± 60.9	54.1 ± 73.5	0.943	0.351
Follow-up (weeks)	46.1 ± 23.5	44.2 ± 22.5	51.4 ± 26.2	0.988	0.328

CHC: chronic hepatitis C; CIR: cirrhosis; BMI: body mass index; ALT: alanine aminotransferase; TBil: total bilirubin; IDUs: intravenous drugs users.

**Table 2:** The virological response of DAAs treatment.

	All (*n* = 54)	CHC (*n* = 39)	CIR (*n* = 15)	Statistics	*p*
Regimen (1/2/3/4/5)	16/17/12/7/2	12/16/4/7/0	4/1/8/0/2	18.823	<0.001
RVR	32/46 (69.6%)	24/32 (75.0%)	8/14 (57.1%)	0.745	0.388
EVR	45/46 (97.8%)	31/31(100%)	14/15 (93.3%)	—	0.326
EOTR	53 (98.1%)	39 (100%)	14 (93.3%)	—	0.278
SVR12	51 (94.4%)	38 (97.4%)	13 (86.7%)	—	0.183
Relapse	3 (5.6%)	1 (2.6%)	2 (13.3%)	—	0.183

CHC: chronic hepatitis C; CIR: cirrhosis; Regimen: 1 = SOF + RBV, 2 = SOF + DCV, 3 = SOF + DCV + RBV, 4 = SOF/VEL, and 5 = SOF/VEL + RBV, 12 weeks or 24 weeks; RVR: rapid virological response; EVR: early virological response; EOTR: end of treatment response; SVR12: sustained virological response 12 weeks after the end of treatment.

**Table 3:** The biochemical response of DAAs treatment.

	All (*n* = 54)	CHC (*n* = 39)	CIR (*n* = 15)	Statistics	*p*
ALT0 (IU/L)	91.6 ± 68.0	99.6 ± 75.6	70.0 ± 35.1	1.841	0.072
Baseline ALT abnormality	36 (66.7%)	26 (66.7%)	10 (66.7%)	0.000	1.000
ALT normality					
W4	44 (81.5%)	35 (89.7%)	9 (60.0%)	4.533	0.033
W12	49 (90.7%)	36 (92.3%)	13 (86.7%)	0.014	0.907
EOT	48 (88.9%)	36 (92.3%)	12 (80.0%)	0.649	0.420
F12	47 (87.0%)	37 (94.9%)	10 (66.7%)	5.343	0.021
TBil0 (*μ*mol/L)	13.8 ± 7.4	11.2 ± 4.6	20.3 ± 9.4	3.306	0.005
ΔTB4_0	1.1 ± 5.0	1.1 ± 4.6	1.3 ± 5.9	0.154	0.878
ΔTB12_0	−0.6 ± 6.1	−0.4 ± 4.2	−1.1 ± 9.2	0.238	0.815
ΔTB_EOT_0	−1.4 ± 4.5	−1.3 ± 3.6	−1.9 ± 6.2	0.334	0.743
ΔTB_F12_0	−2.4 ± 6.6	−3.4 ± 5.0	0.2 ± 9.5	1.124	0.285
eGFR0 (ml/min.1.73 m^2^)	107.3 ± 19.0	107.0 ± 21.8	108.3 ± 9.8	0.162	0.872
ΔeGFR4_0	2.4 ± 11.1	4.6 ± 12.8	−2.2 ± 4.7	1.445	0.163
ΔeGFR12_0	0.5 ± 11.3	1.6 ± 12.8	−1.9 ± 7.1	0.903	0.372
ΔeGFR_EOT_0	2.3 ± 11.9	2.8 ± 13.6	1.3 ± 7.1	0.324	0.748
ΔeGFR_F12_0	0.0 ± 12.4	−0.8 ± 13.9	1.6 ± 8.9	0.503	0.618

CHC: chronic hepatitis C; CIR: cirrhosis; BMI: body mass index; ALT: alanine aminotransferase; TBil: total bilirubin; eGFR: estimated glomerular filtration rate; W4: week 4 of treatment; W12: week 12 of treatment; EOT: end of treatment; F12: 12 weeks after end of treatment; ALT0: ALT at baseline (same for TBil0 and eGFR0); ΔTB4_0 or ΔeGFR4_0: changes of TBil or eGFR from baseline to week 4 (TB4–TB0, or eGFR4–eGFR0); ΔTB12_0 or ΔeGFR12_0: changes of TBil or eGFR from baseline to week 12; ΔTB_EOT_0 or ΔeGFR_EOT_0: changes of TBil or eGFR from baseline to end of treatment; ΔTB_F12_0 or ΔeGFR_F12_0: changes of TBil or eGFR from baseline to 12 weeks after end of treatment.

## Data Availability

The data used to support the findings of this study are included within the article.
